# Estimation in multi‐arm two‐stage trials with treatment selection and time‐to‐event endpoint

**DOI:** 10.1002/sim.7367

**Published:** 2017-06-13

**Authors:** Matthias Brückner, Andrew Titman, Thomas Jaki

**Affiliations:** ^1^ Department of Mathematics and Statistics Lancaster University Lancaster LA1 4YF U.K.

**Keywords:** multi‐arm trial, time‐to‐event, adaptive design, bias, empirical Bayes

## Abstract

We consider estimation of treatment effects in two‐stage adaptive multi‐arm trials with a common control. The best treatment is selected at interim, and the primary endpoint is modeled via a Cox proportional hazards model. The maximum partial‐likelihood estimator of the log hazard ratio of the selected treatment will overestimate the true treatment effect in this case. Several methods for reducing the selection bias have been proposed for normal endpoints, including an iterative method based on the estimated conditional selection biases and a shrinkage approach based on empirical Bayes theory. We adapt these methods to time‐to‐event data and compare the bias and mean squared error of all methods in an extensive simulation study and apply the proposed methods to reconstructed data from the FOCUS trial. We find that all methods tend to overcorrect the bias, and only the shrinkage methods can reduce the mean squared error. © 2017 The Authors. *Statistics in Medicine* Published by John Wiley & Sons Ltd.

## 1 Introduction

Multi‐arm trials, which compare multiple treatment arms to a common control group, are more efficient than traditional two‐arm designs as only a single control group is used 1. Additional efficiency gains can be achieved by selecting treatments at one or more interim analyses 2. Most of the recent research in this area focuses on how such studies are designed for a normally distributed endpoint (e.g., 3, 4, 5, 6). Approaches that are applicable to non‐normal and particularly time‐to‐event endpoints are less frequent in contrast (e.g., 7, 8, 9, 10), although many examples of multi‐arm trials with survival endpoints, such as the three‐arm SANAD trial in generalized and unclassifiable epilepsy 11, the four‐arm STAMPEDE trial in prostate cancer 12, or the five‐arm FOCUS trial in poor prognosis advanced colorectal cancer 13, exist.

Adaptive treatment selection at an interim analysis increases the chance of a successful trial and avoids wasting resources on unpromising treatments 1. It is well known, however, that selecting promising treatments based on the observed data leads to overestimation of the treatment effect in the selected treatment arms and an underestimation of the treatment effect in the dropped treatment arms 14. A variety of different strategies to remove or reduce the bias has been developed for normal data. The uniformly minimum variance conditionally unbiased estimator (UMVCUE) in a two‐stage design when selecting the best treatment was first derived by Cohen and Sackrowitz 15 and extended to more general selection rules in 16. A simple iterative method for calculating a bias‐corrected estimate based on the estimated conditional selection bias was proposed by Stallard and Todd 17. An extension of the shrinkage estimator of Hwang 18 to two‐stage adaptive designs has been proposed by Carreras and Brannath 19. They show that the shrinkage estimator has smaller Bayes risk than the maximum likelihood estimator (MLE) and also a smaller mean squared error (MSE) in many scenarios.

Common to the methods for normal data is that they assume equal variance between treatment arms and do not consider the control group, because in the normal case, the conditional selection bias of the MLE is the same with or without a control group (although the equal variance assumption has been relaxed for the UMVCUE in recent work 20, which also explicitly accounts for a shared control group). However, in the context of survival trials and for the estimation of log hazard ratios (log‐HRs), a control group is required. The challenge thereby is that a control group, which is common to all treatment groups, induces a correlation among the log‐HR estimators. Moreover, the assumption of equal and known variances is unrealistic in the survival setting as the variances depend on the number of observed events. In this paper, we therefore adapt the method of Stallard and Todd 17 to time‐to‐event endpoints and consider shrinkage estimators in this context. In an extensive simulation study, we then compare the bias and MSE of these estimators and analyze reconstructed data from the FOCUS trial.

In Section 2.2, we derive explicit formulas for the selection bias of the MLE in the selected, as well as in the dropped treatment arms under a Cox proportional hazards model, when the treatment with the largest observed effect at an interim analysis is selected. We derive two shrinkage estimators in Section 2.3 and adapt the iterative Stallard–Todd method to survival data in Section 2.4. The simulation study in Section 3 compares the bias and MSE of these methods for different number of treatment groups, allocation ratios, and total number of events. An analysis of reconstructed data from the FOCUS trial is presented in Section 4 before we conclude with a discussion.

## 2 Methods

We consider a two‐stage design where the best treatment, that is, the treatment with the smallest estimated log‐HR, is selected at the interim analysis. Let *S* be the index of the selected treatment. Note that *S* is a random variable depending on the Stage 1 data.

### 2.1 Model

Consider K⩾2 treatment groups and a common control group. Denote the total sample size by *n*, and suppose patients are allocated to group *k* in Stage 1 with probability *p*_*k*_ (*k* = 0, …, *K*). Here, the index *k*=0 represents the control group. Assuming proportional hazards between each treatment group and the control group, we consider the following Cox proportional hazards model:

(1) $$\lambda_{j}(s)=\lambda_{0}(s)\exp\left(\beta^{T}Z_{j}\right)\;j=1,\ldots ,n,$$

where *β* is the vector of log‐HRs and *Z*_*j*_=(*Z*_1*j*_, …, *Z*_*Kj*_) is the vector of binary treatment indicators, that is, *Z*_*kj*_=1 if patient *j* is in group *k* and 0, otherwise (*k* = 1 …, *K*, *j* = 1, …, *n*). The joint model in Eq. (1) is equivalent to *K* separate Cox models with the same baseline hazard function *λ*_0_, which is the hazard in the common control group. In a sequential survival trial with more than one analysis time point, we need to distinguish between the calendar time, measured from the start of the trial, and the individual survival time of each patient. Associated with each patient is an entry time *R*_*j*_, measured from the start of the trial, a survival time *T*_*j*_, and a censoring time *C*_*j*_, both measured from entry of the patient into the trial. At each calendar time *t*, only the minimum $Y_{j}(t)=\min(T_{j},C_{j},t-R_{j})$ and the censoring indicator $\Delta_{j}(t)=I\{T_{j}⩽\min(C_{j},t-R_{j})\}$ can be observed. The observed data of all patients at calendar time *t* are given by

$$D(t)=\left\{\left(Y_{j}(t),\Delta_{j}(t),R_{j},Z_{j}\right),j=1,\ldots ,n(t)\right\},$$

where $n(t)=\sum_{j}I\left\{R_{j}⩽t\right\}$ is the number of patients recruited by calendar time *t*.

### 2.2 Maximum likelihood estimator

The MLE of *β* in Eq. (1) at calendar time *t* is denoted by ${\hat{\beta }}^{\text{MLE}}(t)=({\hat{\beta }}_{1}^{\text{MLE}}(t),\ldots ,{\hat{\beta }}_{K}^{\text{MLE}}(t))$ and is obtained by maximizing the partial‐likelihood of the Cox model based on the data *D*(*t*). In practice, interim and final analyses are usually not scheduled at fixed calendar times but after a certain number of events have been observed. Let *d*_*j*_ be the total number of events of the *j*‐th analysis and *d*_*kj*_ the number of events in the *k*‐th group at the *j*‐th analysis, such that *d*_*j*_=*d*_*j*0_ + … + *d*_*jK*_ (*k* = 0, …, *K*, *j* = 1, 2). Denote by ${\hat{\beta }}^{\text{MLE},j}$ the vector of estimated log‐HRs ${\hat{\beta }}_{k}^{\text{MLE},j}$ (*k* = 1, …, *K*, *j* = 1, 2) at the interim and final analyses, respectively. The estimated log‐HR in the selected treatment group at the interim analysis is ${\hat{\beta }}_{S}^{\text{MLE},1}=\min_{k=1,\ldots ,K}\{{\hat{\beta }}_{k}^{\text{MLE},1}\}$. The true treatment effect *β*_*S*_ of the selected treatment is a random variable, and in general, $\beta_{S}\neq \min_{k}\beta_{k}$. We say that an estimator ${\hat{\beta }}_{S}^{\text{MLE},j}$ of *β*_*S*_ at Stage *j* is unbiased if $E[{\hat{\beta }}_{S}^{\text{MLE},j}]=E[\beta_{S}]$ (*j*=1,2) 15.

The MLEs are correlated because of the common control group. If *β*≈0 and the censoring time *C* is stochastically independent from *Z*, we show in Section A.1 in the Appendix that the correlation *ρ*_*kl*_ between ${\hat{\beta }}_{k}^{\text{MLE}\;,j}$ and ${\hat{\beta }}_{l}^{\text{MLE}\;,j}$ is approximately

$$\frac{\sqrt{p_{k}p_{l}}}{\sqrt{p_{0}+p_{k}}\sqrt{p_{0}+p_{l}}}>0\;(1⩽k,l⩽K).$$

Note that when the size of the common control group decreases, the correlation of the MLEs increases. In the special case $p_{0}=1-\sum_{k=1}^{K}p_{k}=1-Kp$, where all treatment groups are the same size, the correlation *ρ* between any two log‐HR estimators at the interim analysis is approximately (1−*p*_0_)/(1+(*K*−1)*p*_0_).

The derivation of the selection bias is based on the joint asymptotic distribution of the vectors ${\hat{\beta }}^{\text{MLE}\;,1}$ and ${\hat{\beta }}^{\text{MLE}\;,2}$. We have approximately

$${\left({\hat{\beta }}^{\text{MLE}\;,1},{\hat{\beta }}^{\text{MLE}\;,2}\right)}^{T}∼N\left({\left(\beta ,\beta \right)}^{T},\left(\begin{array}{cc} \Sigma_{1} & \Sigma_{2}\\ \Sigma_{2} & \Sigma_{2} \end{array}\right)\right),$$

where Σ_*j*_ is the asymptotic covariance matrix of ${\hat{\beta }}^{\text{MLE}\;,j}$ (*j*=1,2) (see Section A.1 in the Appendix).

#### 2.2.1 Estimation in a two‐stage design

Denote by ${\hat{\delta }}_{S}^{\text{MLE}}$ the increment of the MLE based on all data observed after the interim analysis (including events observed in Stage 2, from patients recruited in Stage 1). This is an (asymptotically) unbiased estimator of the true log‐HR, which is inefficient, because it ignores all of the Stage 1 data. Formally, ${\hat{\delta }}_{S}^{\text{MLE}}$ can be defined via the score process *U* of the Cox model and the corresponding Fisher information I conditional on the selection *S*. Asymptotically *U*_2*S*_−*U*_1*S*_ and *U*_1*S*_ are stochastically independent, because of the independent increments property of *U*. Thus, the increment of the MLE given by

$${\hat{\delta }}_{S}^{\text{MLE}}=\frac{U_{2S}-U_{1S}}{I_{2S}-I_{1S}},$$

is asymptotically independent from ${\hat{\beta }}_{S}^{\text{MLE}\;,1}$ conditionally on *S*. At the final analysis, the MLE of the selected treatment can be defined in two different, but asymptotically equivalent, ways, either by ${\hat{\beta }}^{\text{MLE}\;,2}$, that is, from all data accrued until the final analysis or by a weighted sum of the first stage MLE and the increment from first to second stage, that is,

$${\hat{\beta }}_{S}^{\text{MLE}\;,1,\delta }:=w{\hat{\beta }}_{S}^{\text{MLE}\;,1}+(1-w){\hat{\delta }}_{S}^{\text{MLE}\;},$$

where *w*∈[0,1] is a pre‐specified constant. We will consider both estimators in our simulation study. Usually, *w* will be equal to the information fraction at the planned time of the interim analysis, that is, *w*=*d*_1_/*d*_2_.

#### 2.2.2 Selection bias and mean squared error

The bias of the MLE of the selected treatment at analysis *j* can be written as

$$\text{Bias}\left({\hat{\beta }}_{S}^{\text{MLE}\;,j}\right)=E\left[{\hat{\beta }}_{S}^{\text{MLE}\;,j}-\beta_{S}\right]=\overset{K}{\underset{k=1}{\sum }}E\left[{\hat{\beta }}_{k}^{\text{MLE}\;,j}-\beta_{k}|S=k\right]P(S=k).$$

The true treatment effect in the selected treatment arm is overestimated, when the best performing treatment arm is selected, because

$$E\left[\underset{k=1,\ldots ,K}{\min}{\hat{\beta }}_{k}^{\text{MLE}\;,1}\right]⩽\underset{k=1,\ldots ,K}{\min}E\left[{\hat{\beta }}_{k}^{\text{MLE}\;,1}\right]=\underset{k=1,\ldots ,K}{\min}E[\beta_{k}]⩽E[\beta_{S}].$$

One can see that the true treatment effect in the dropped treatment arms is underestimated.

For normal means, the selection bias is maximal if all means are equal 19. Their result and its proof still hold asymptotically for the estimated log‐HRs when the signs are reversed; that is, for given variances and correlation, the absolute value of the bias is maximal if all true log‐HRs are equal. We obtain a lower bound

$$C_{j}(\Sigma_{j})=E\underset{k=1,\ldots ,K}{\min}\left\{{\hat{\beta }}_{k}^{\text{MLE}\;,j}-\beta_{k}\right\}$$

for the selection bias of ${\hat{\beta }}_{S}^{\text{MLE}\;,j}$ depending on the asymptotic covariance matrix Σ_*j*_. This bound is attained when the true log‐HRs are all equal. Because the correlations are always nonnegative in our setting, it is an immediate consequence of Slepian's lemma 21 that for fixed variances, the lower bound *C*_*j*_(Σ_*j*_) is minimal when all correlations are 0. This corresponds to a situation where every treatment group has its own independent control group.

There are two possible ways of defining the MSE, because *β*_*S*_ is a random variable. First, the MSE of predicting *β*_*S*_

$$E\left[{\left({\hat{\beta }}_{S}^{\text{MLE}\;,j}-\beta_{S}\right)}^{2}\right]=\mathrm{Var}\left({\hat{\beta }}_{S}^{\text{MLE}\;,j}-\beta_{S}\right)+\text{Bias}{\left({\hat{\beta }}_{S}^{\text{MLE}\;,j}\right)}^{2},$$

which is the definition that we will use in our simulation study, or alternatively the MSE of estimating *E*[*β*_*S*_]:

$$E\left[{\left({\hat{\beta }}_{S}^{\text{MLE}\;,j}-E[\beta_{S}]\right)}^{2}\right]=\mathrm{Var}\left({\hat{\beta }}_{S}^{\text{MLE}\;,j}\right)+\text{Bias}{\left({\hat{\beta }}_{S}^{\text{MLE}\;,j}\right)}^{2}.$$

Note that while the bias does not depend on the correlation in the normal case, this is not the case for the variance. The MSE is always larger with control group than without a control group:

$$E\left[{\left(X_{S}-X_{0}-(\theta_{S}-\theta_{0})\right)}^{2}\right]=E\left[{\left(X_{S}-\theta_{S}\right)}^{2}\right]+\mathrm{Var}(X_{0})>E\left[{\left(X_{S}-\theta_{S}\right)}^{2}\right],$$

where *X*_*k*_ is the sample mean and *θ*_*k*_ is the true mean in group *k* = 0, …, *K*.

#### 2.2.3 Conditional selection bias

We will now give explicit expressions for the selection probabilities and the conditional selection biases at the interim and final analyses. The detailed derivations can be found in Section B.1 in the Appendix.

Let ${\hat{\beta }}_{-k}^{\text{MLE}\;,1}$ be the vector ${\hat{\beta }}^{\text{MLE}\;,1}$ with the *k*‐th element removed. Denote by

$$S_{\beta_{-k}(x),\Sigma_{-k}(x)}(x)=\int_{x}^{\infty }\cdots \int_{x}^{\infty }\phi_{\beta_{-k}(x),\Sigma_{-k}(x)}(y_{1},\ldots ,y_{K-1})dy_{1}\cdots dy_{K-1}$$

the survival function of the (*K*−1)‐dimensional normal distribution with mean *β*_−*k*_(*x*) and covariance matrix Σ_−*k*_(*x*), where *β*_−*k*_(*x*) is the conditional mean and Σ_−*k*_(*x*) is the conditional covariance matrix of ${\hat{\beta }}_{-k}^{\text{MLE}\;,1}$ given ${\hat{\beta }}_{k}^{\text{MLE}\;,1}=x$, respectively. The probability of selecting treatment *k* is given by

$$P(S=k)=\int_{-\infty }^{\infty }S_{\beta_{-k}(x),\Sigma_{-k}(x)}(x)\phi \left(\frac{x-\beta_{k}}{\sigma_{1k}}\right)\sigma_{1k}^{-1}dx,$$

where $\sigma_{1k}=\sqrt{\Sigma_{1kk}}$. At the interim analysis, the conditional selection bias ${\text{cb}}_{1k}^{+}$ of ${\hat{\beta }}_{k}^{\text{MLE}\;,1}$ conditional on {*S*=*k*} is

(2) $$\begin{array}{c} {\text{cb}}_{1k}^{+}=E\left[{\hat{\beta }}_{k}^{\text{MLE}\;,1}|S=k\right]-\beta_{k}=\frac{\int_{-\infty }^{\infty }(x-\beta_{k})S_{\beta_{-k}(x),\Sigma_{-k}(x)}(x)\phi \left(\frac{x-\beta_{k}}{\sigma_{1k}}\right)\sigma_{1k}^{-1}dx}{\int_{-\infty }^{\infty }S_{\beta_{-k}(x),\Sigma_{-k}(x)}(x)\phi \left(\frac{x-\beta_{k}}{\sigma_{1k}}\right)\sigma_{1k}^{-1}dx}. \end{array}$$

In the dropped treatment arm, the MLE is biased in the opposite direction; that is, the treatment effect is underestimated, and the bias ${\text{cb}}_{1k}^{-}$ of ${\hat{\beta }}_{k}^{\text{MLE}\;,1}$ given {*S*≠*k*} is

(3) $${\text{cb}}_{1k}^{-}=E\left[{\hat{\beta }}_{k}^{\text{MLE}\;,1}|S\neq k\right]-\beta_{k}=-\left(E\left[{\hat{\beta }}_{k}^{\text{MLE}\;,1}|S=k\right]-\beta_{k}\right)\frac{P(S=k)}{1-P(S=k)}.$$

We derive now an explicit formula for the conditional selection bias at the final analysis. Let $u_{k}=\Sigma_{2k.}\Sigma_{1}^{-1}$, where Σ_2*k*·_ is the *k*‐th row of the matrix Σ_2_. Then, the conditional selection bias at the final analysis can be written as

(4) $${\text{cb}}_{2k}^{+}=\overset{K}{\underset{l=1}{\sum }}u_{kl}v_{kl},$$

where

$$v_{kl}=\left\{\begin{array}{cc} E\left[{\hat{\beta }}_{l}^{\text{MLE}\;,1}|S\neq l\right]-\beta_{l} & \;\text{for}l\neq k\\ E\left[{\hat{\beta }}_{k}^{\text{MLE}\;,1}|S=k\right]-\beta_{k} & \;\text{for}l=k. \end{array}\right.$$

In the dropped treatment arm, analogously to Eq. (3),

(5) $${\text{cb}}_{2k}^{-}=-{\text{cb}}_{2k}^{+}\frac{P(S=k)}{1-P(S=k)}.$$

We have shown in Section 2.2.2 that the absolute value of the bias is maximal when the correlation is 0; that is, each treatment group has its own independent control group. In this case, Eqs (4) and (5) reduce to

$${\text{cb}}_{2k}^{+}=\frac{\sigma_{2k}^{2}}{\sigma_{1k}^{2}}{\text{cb}}_{1k}^{+}$$

and

$${\text{cb}}_{2k}^{-}=\frac{\sigma_{2k}^{2}}{\sigma_{1k}^{2}}{\text{cb}}_{1k}^{-},$$

respectively. Thus,

(6) $$\frac{{\text{cb}}_{2k}^{+}}{{\text{cb}}_{1k}^{+}}=\frac{{\text{cb}}_{2k}^{-}}{{\text{cb}}_{1k}^{-}}=\frac{\sigma_{2k}^{2}}{\sigma_{1k}^{2}}\approx \frac{d_{1k}}{d_{2k}}.$$

Thus, each additional event observed after the interim analysis reduces the conditional bias, and the bias at the final analysis will be smaller than at the interim analysis. We also see that only the total number of observed events matters and not whether the event comes from a patient recruited before or after the interim analysis. This is especially interesting in the dropped treatment arms, because it quantifies the bias reduction that we can expect, when continuing follow‐up until the final analysis. In our simulation study in Section 3, we see that this relation also holds approximately in the case of a common control group. This result is analogous to the normal case, for example 22.

In a two‐arm trial with a single treatment group, recruitment may be stopped early at the interim analysis for the lack of benefit, when the estimated log‐HR is larger than some pre‐specified threshold *c*, but follow‐up is continued until the final analysis. The selection bias in this case can be obtained as a special case by setting $K=2,{\hat{\beta }}_{2}^{\text{MLE}\;,1}=c$, and letting $\sigma_{12}\to 0$. This leads to

$${\text{cb}}_{j1}^{-}=E\left[{\hat{\beta }}_{1}^{\text{MLE}\;,j}|{\hat{\beta }}_{1}^{\text{MLE}\;,1}>c\right]\beta_{1}=\frac{\sigma_{j1}^{2}}{\sigma_{11}}\frac{\phi \left(\frac{c-\beta_{1}}{\sigma_{11}}\right)}{1-\Phi \left(\frac{c-\beta_{1}}{\sigma_{11}}\right)}.$$

We see that Eq. (6) still holds; that is, additional events reduce the conditional selection bias. This confirms the simulation study results of 23.

### 2.3 Shrinkage estimator

We consider two different approaches to deriving the shrinkage estimator. The first approach follows closely the original derivation of 18, by considering the posterior mean of the selected treatment when the covariance matrix of the data is known, but arbitrary, and then replacing the prior mean and variance by their sample estimates. The second approach first scales the estimators by the square root of their covariance matrix to obtain independent estimators with unit variances and then rescales the shrinkage estimates back to the original scale.

In the normal case with known and equal variances and four or more treatment groups, the shrinkage estimator can be derived as an empirical Bayes estimator 24. Let *X*_*k*_ be the sample mean and *θ*_*i*_ the true mean in group *k* ( k=1,…,K⩾4). We assume normal priors for the true means *θ*_*k*_∼*N*(*μ*,*τ* ^2^) and that the sample mean *X*_*k*_ conditional on *θ*_*k*_ is normally distributed with mean *θ*_*k*_ and known variance *σ* ^2^ (*k* = 1, …, *K*). The posterior mean of *θ*_*k*_ conditional on *X*_*k*_ is given by (1−*C*)*μ*+*C* *X*_*k*_ and the posterior variance by *σ* ^2^ *C*, where *C* = *τ* ^2^/(*τ* ^2^+*σ* ^2^). Thus, the posterior mean of *θ*_*S*_ is *E*[*θ*_*S*_|*X*_1_, …, *X*_*K*_] = (1−*C*)*μ*+*C* *X*_*S*_. To obtain an estimator of the posterior mean of *θ*_*S*_, the unknown quantities *μ* and *C* are estimated by the overall sample mean $\bar{X}$ and

$$Ĉ=1-\frac{(K-3)\sigma^{2}}{\overset{K}{\underset{k=1}{\sum }}{\left(X_{k}-\bar{X}\right)}^{2}},$$

respectively. Ĉ is an unbiased estimator of *C* 24. Because C⩾0, the estimator Ĉ is replaced by ${Ĉ}^{+}=\max(Ĉ,0)$. Therefore, the shrinkage estimator is given by

$$Q=(1-{Ĉ}^{+})\bar{X}+{Ĉ}^{+}X.$$

The aforementioned estimators are only defined for K⩾4. The best linear unbiased predictor can be used instead when K⩽3. The best linear unbiased predictor is the same as the empirical Bayes estimator but with *K*−3 replaced by *K*−1^19^.

#### 2.3.1 Shrinkage estimator for the log hazard ratios

The extension from independent equal variances to the general case of an arbitrary covariance matrix is straightforward. In order to simplify the notation, we write $\hat{\beta }$ in the following for the MLE at Stage *j* instead of ${\hat{\beta }}^{\text{MLE}\;,j}$. Assume now

(7) $$\begin{array}{ccc} \beta & ∼N(\mu ,\tau^{2}I) & \\ \hat{\beta }|\beta & ∼N(\beta ,\Sigma ), \end{array}$$

where *I* is the *K*×*K* identity matrix and the Σ the covariance matrix, which we assume to be known for the moment. Note that we still put independent priors on the true treatment effects. The posterior mean of *β* given the data $\hat{\beta }$ is

$${\left(\tau^{-2}I+\Sigma^{-1}\right)}^{-1}\left(\tau^{-2}\mu +\Sigma^{-1}\hat{\beta }\right)=C\hat{\beta }+(I-C)\mu ,$$

where the shrinkage factor *C* is now the matrix *I*−Σ(*τ* ^2^ *I*+Σ)^−1^. The marginal covariance matrix of $\hat{\beta }$ is *τ* ^2^ *I*+Σ. Because Σ is symmetric, there exists an orthogonal matrix *U* and a diagonal matrix *D* such that $D=U\Sigma U^{\prime }$. Thus, $U\hat{\beta }$ is a vector of independent normal distributed variables. The marginal covariance matrix of $U\hat{\beta }$ is *τ* ^2^ *I*+*D*. The following iterative procedure described by Morris 24 to calculate the MLE ${\hat{\tau }}^{2}$ of *τ* ^2^ can now be applied to the transformed data $U\hat{\beta }$:

1. Start with an initial guess of ${\hat{\tau }}^{2}$.
2. Define weights $w_{k}={\left({\hat{\tau }}^{2}+D_{kk}^{2}\right)}^{-1}$ (*k* = 1, …, *K*).
3. Estimate *τ* ^2^ by
  $${\hat{\tau }}^{2}=\frac{\overset{K}{\underset{k=1}{\sum }}w_{k}\left\{{\left({\hat{\beta }}_{k}-\bar{\beta }\right)}^{2}-D_{kk}^{2}\right\}}{\overset{K}{\underset{k=1}{\sum }}w_{k}}.$$
4. If ${\hat{\tau }}^{2}<0$, set ${\hat{\tau }}^{2}=0$.
5. Repeat Steps 2 and 4 until convergence.

As Σ is unknown, we apply this iterative method with the diagonal matrix $\hat{D}=Û\hat{\Sigma }{Û}^{\prime }$ instead of *D*, where $\hat{\Sigma }$ is an estimator of the unknown covariance matrix Σ and Û is an orthogonal matrix to diagonalize $\hat{\Sigma }$. We can now define an estimator of the matrix *C* by

$$Ĉ=I-\hat{\Sigma }{\left({\hat{\tau }}^{2}I+\hat{\Sigma }\right)}^{-1}.$$

Similarly as before, we replace the unknown prior mean *μ* by the overall log‐HR $\bar{\beta }$, which is calculated by pooling all treatment groups together and fitting a Cox proportional hazards model to the pooled data. The shrinkage estimator is defined by

$${\hat{\beta }}^{\text{EB}}=Ĉ\hat{\beta }+(I-Ĉ)\bar{\beta }.$$

The shrinkage estimator of the selected treatment is ${\hat{\beta }}_{S}^{\text{EB}}$. If ${\hat{\tau }}^{2}=0$, then Ĉ=0, and the shrinkage estimator is equal to the overall log‐HR $\bar{\beta }$. We will call this the EB shrinkage method.

We could have diagonalized with the orthogonal transformation *U* from the beginning; that is, we could have used *U* *θ* and $U\hat{\beta }$, applied the shrinkage, and then transformed back to the original coordinates. This would have led to the shrinkage factor:

$$\tilde{C}=U^{\prime }U-U^{\prime }D{\left(\tau^{2}I+D\right)}^{-1}U,$$

which is exactly the same as *C*, because *U* is orthogonal.

#### 2.3.2 Shrinkage estimator of the scaled log hazard ratios

Instead of only diagonalizing $\hat{\Sigma }$, we standardize it to the identity matrix and then apply the method of the independent equal variances case. This leads to an alternative shrinkage estimator. Consider the problem of shrinkage towards 0 when Σ is known. We start by transforming the vector $\hat{\beta }$ with covariance matrix Σ to a vector *β* ^∗^ with independent components and unit variances. Define the scaled MLEs:

$${\hat{\beta }}^{*}=\Sigma^{-1/2}\hat{\beta }∼N(\Sigma^{-1/2}\beta ,I).$$

When we use a *N*(*μ* ^∗^,*τ* ^∗2^ *I*) prior on the scaled treatment effects Σ^−1/2^ *β*, we know from the independent case with equal variances that the empirical Bayes estimator is given by ${Ĉ}^{+}{\hat{\beta }}^{*}$, where

$${Ĉ}^{+}=\max\left(1-\frac{K-3}{\overset{K}{\underset{k=1}{\sum }}\overset{*2}{\underset{k}{\hat{\beta }}}},0\right).$$

We have

$$\overset{K}{\underset{k=1}{\sum }}{\hat{\beta }}_{k}^{*2}={\hat{\beta }}^{T}\Sigma^{-1}\hat{\beta }.$$

The right hand side is the Wald test statistic for testing equality of all treatment effects, which is asymptotically equivalent to the log‐rank test statistic in the Cox model in Eq. (1). When shrinking towards the overall log‐HR $\bar{\beta }$, we take the log‐rank test statistic *Z*, which directly compares the treatment groups, that is, without the control group and treatment group 1 being the new baseline, instead. Therefore,

$${Ĉ}^{+}=\max\left(1-\frac{K-3}{Z},0\right).$$

As in the normal case, we replace *K*−3 by *K*−1 if *K*<4. The log‐rank test statistic *Z* is asymptotically $\chi_{K-1}^{2}$ distributed. This is the justification to consider the shrinkage estimator:

$${\hat{\beta }}^{\text{LR}}={Ĉ}^{+}\hat{\beta }+\left(1-{Ĉ}^{+}\right)\bar{\beta },$$

which we will call the LR shrinkage method.

#### 2.3.3 Two‐stage shrinkage estimators

We define a two‐stage estimator similar to the two‐stage shrinkage estimator proposed by Carreras and Brannath 19 using the increment of the MLE:

(8) $${\hat{\beta }}_{S}^{*,1,\delta }:=w{\hat{\beta }}_{S}^{*,1}+(1-w){\hat{\delta }}_{S}^{\text{MLE}\;},$$

where ${\hat{\beta }}_{S}^{*,1}$ is one of the two shrinkage estimators at the interim analysis defined previously.

#### 2.3.4 Confidence intervals

Our focus is on point estimation. Construction of correct confidence intervals is difficult and a topic of ongoing research. No adequate methods for constructing confidence intervals in this setting are available apart from bootstrap resampling. However, bootstrap confidence intervals cannot properly account for the variability introduced by the selection process, because the selection in a resampled data set may be different from the decision in the original data set, but the decision to stop recruitment in the dropped treatment arms cannot be reversed in retrospect. Despite this, such intervals are useful to compare the relative merits of the estimators. We use a bootstrap approach conditional on the selection *S*_0_ in the original data set similar to 25:

1. Resample data accrued until final analysis;
2. Determine calendar time *t* ^∗^ of interim analysis in resampled data set;
3. Obtain Stage 1 data from resampled data set, and if necessary, recensor event times of overrunning patients at *t* ^∗^;
4. Calculate Stage 1 MLEs ${\tilde{\beta }}_{k}$ (*k*=1,…,*K*) from Stage 1 data;
5. If ${\tilde{\beta }}_{S_{0}}=\min_{k}{\tilde{\beta }}_{k}$, calculate bias‐corrected estimates.

These steps should be repeated until a large enough number of estimates have been produced in order to calculate empirical quantiles of the sampling distributions with the desired accuracy. Note that the interim analysis in each resampled data set is conducted at the same information fraction as in the original data set, although this may correspond to a different number of events and calendar time.

### 2.4 Stallard–Todd estimator

The bias‐corrected estimator of Stallard and Todd 17, 26 is the solution ${\hat{\beta }}^{ST,1}$ of

(9) $$\tilde{\beta }={\hat{\beta }}^{\text{MLE}\;,1}-b(\tilde{\beta }),$$

where $b(\tilde{\beta })=(b_{1}(\tilde{\beta }),\ldots ,b_{K}(\tilde{\beta }))$ is an estimate of the vector of conditional biases when the true log‐HR is $\tilde{\beta }$ (Section 2.2.3). A simple fixed point iteration can be used to find ${\hat{\beta }}^{ST,1}$:

1. ${\tilde{\beta }}_{0}={\hat{\beta }}^{\text{MLE}\;,1}$;
2. ${\tilde{\beta }}_{n+1}={\hat{\beta }}^{\text{MLE}\;,1}-b({\tilde{\beta }}_{n})$;
3. Repeat Step 2 until convergence.

The conditional biases depend on the unknown covariance matrix of ${\hat{\beta }}^{\text{MLE}\;,1}$ (Section 2.2.3), which we replace with the estimator ${\hat{\Sigma }}_{1}$ from the Cox model. The estimation of the vector $b(\tilde{\beta })$ is computationally complex, because it requires the numeric evaluation of integrals of multivariate cumulative distribution functions. Transforming the vector ${\hat{\beta }}^{\text{MLE}\;,1}$ in the same way as in Section 2.3.2 and then solving Eq. (9) with the identity matrix as covariance matrix does not work, because these solutions are in general not solutions of the original equation.

We have defined the Stallard–Todd estimator only at the interim analysis. A similar definition is possible at the final analysis using the bias formulas derived in Section 2.2.3, that is solving Eq. (9) with ${\hat{\beta }}^{\text{MLE}\;,2}$ instead of ${\hat{\beta }}^{\text{MLE}\;,1}$. However, in our simulations, the iterative procedure diverged most of the time. This also happened in the data example in Section 4. This seems to be related to the fact that the selection bias depends on the marginal covariance matrix, but only the conditional covariance matrix given the selection can be estimated at the final analysis. We therefore only use the two‐stage estimator at the final analysis defined by

(10) $${\hat{\beta }}_{S}^{\text{ST},1,\delta }:=w{\hat{\beta }}_{S}^{\text{ST},1}+(1-w){\hat{\delta }}_{S}^{\text{MLE}}.$$

Confidence intervals for the Stallard–Todd estimator can only be obtained by resampling, as its (asymptotic) sampling distribution is not known. Simulations not reported here suggest that asymptotic normality does indeed hold.

### 2.5 Bias‐corrected Kaplan–Meier estimator

The Kaplan–Meier estimator in the selected treatment group will be biased upwards. The Kaplan–Meier estimator in the control group will always be (asymptotically) unbiased. We can define a bias‐corrected Kaplan–Meier estimator based on any of the bias‐corrected estimators ${\hat{\beta }}_{S}^{*}(t)$ of the log‐HR by using the relationship between the survival functions in the control and the treatment groups under the Cox model (Eq. (1)). The bias‐corrected Kaplan–Meier estimator at a time *t* is defined as

$${\hat{F}}_{S}^{*}(s)={\hat{F}}_{0}{\left(s\right)}^{\exp\left({\hat{\beta }}_{S}^{*}(t)\right)}\;s⩽t.$$

In simulations not reported here, we found that the selection bias of the Kaplan–Meier estimator in the selected treatment arm is negligible even when all true treatment effects are equal. We would expect the selection bias to be more pronounced, when selection was performed based on, for example, the estimated survival probability at a specific time instead of the estimated log‐HRs at interim.

## 3 Simulations

We consider three base scenarios in our simulation study, which are similar to those in 19 and cover a range of configurations of true log‐HRs. It is of course impossible to cover all possible configurations in a simulation study.

1. Constant: All hazard ratios (HRs) are equal to 1. *H* *R*_*k*_=1(*k* = 1, …, *K*).
2. Linear: A linear trend from 0.6 to 1: *H* *R*_1_=1,*H* *R*_*k*_−*H* *R*_*k*+1_=0.4/(*K*−1)(*k* = 2, …, *K*).
3. Peak: All but one HRs are 1: *H* *R*_1_=0.6,*H* *R*_*k*_ = 1(*k* = 2, …, *K*).

The baseline hazard was log(2)/12, which corresponds to a median survival time of 12 (months) in the control group. We compare the bias and MSE of all methods for an increasing number of treatment groups *K*. A maximum of 200 patients are allocated to each group. The interim analysis was conducted after (*K*+1)·50 observed events in all treatment groups and control group combined. This amounts to approximately 50 events in each group when the event rates are similar. The final analysis was performed after 200 events in the selected treatment group and control group combined.

**Figure 1:**
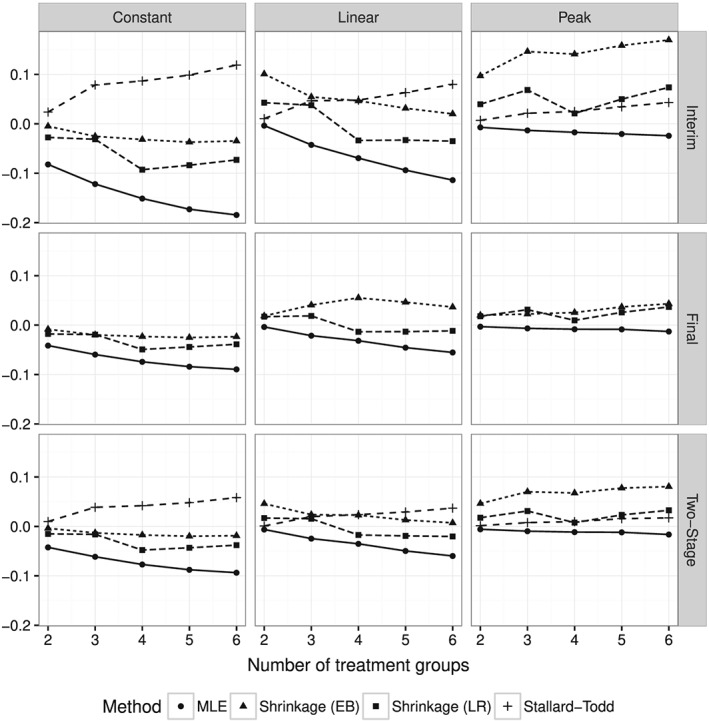
Bias as function of the number of treatment groups in each of the three base scenarios at the interim and final analyses and for the two‐stage estimators. The Stallard–Todd estimator is only defined at the interim analysis and as two‐stage estimator.

**Figure 2:**
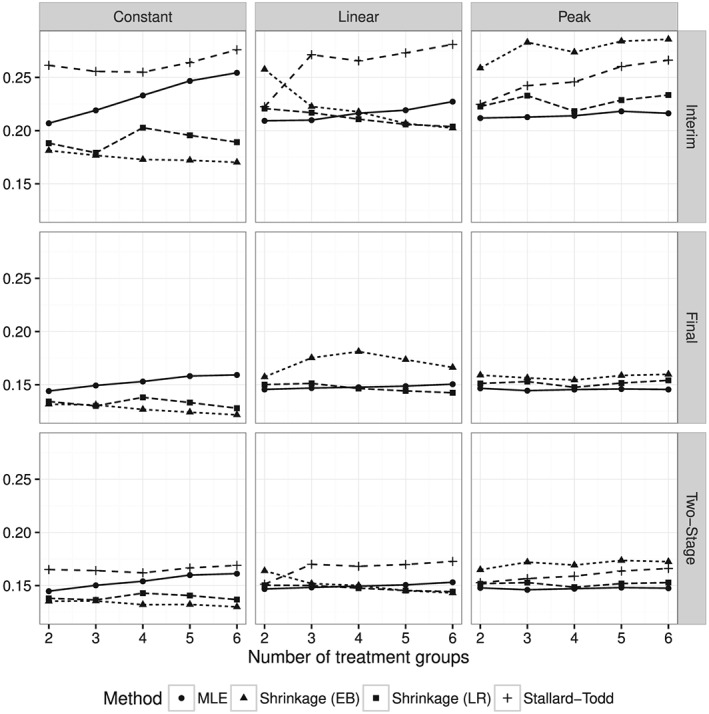
Root mean squared error as function of the number of treatment groups in each of the three base scenarios at the interim and final analyses and for the two‐stage estimators. The Stallard–Todd estimator is only defined at the interim analysis and as two‐stage estimator.

Figure 1 shows the bias in 10^5^ simulations of each method in each of the base scenarios as the number of treatment groups *K* ranges from 2 to 6 at the interim analysis, the final analysis, and for the two‐stage estimators. The corresponding square root of the MSE is shown in Figure 2. We observed some convergence problems of Stallard–Todd estimator, especially in the case *K*=2 with non‐convergence in up to 20*%* of the simulations. In these cases, the MLE was used as a fallback, which slightly increases the bias but reduces the MSE. Overall the results are very similar to those of 19 in the normal case.

The simulation results confirm the theoretical result that the bias of the MLE is maximal, when all treatment effects are equal (“Constant” scenario). Both shrinkage estimators clearly outperform the MLE and the Stallard–Todd method in terms of bias and MSE in this case. In the “Linear” scenario, the LR shrinkage estimator has smaller bias than the MLE, when there are four or more treatment groups and about the same MSE for any number of treatment groups. Both shrinkage estimator win over the Stallard–Todd method except in the *K*=2 case (which is identical to the *K*=2 case in the “Peak” scenario). In the “Peak” scenario, the MLE is practically unbiased and beats all other methods in terms of bias and MSE. However, the LR shrinkage estimator is still very close to the MLE and has a smaller MSE than the Stallard–Todd estimator.

**Figure 3:**
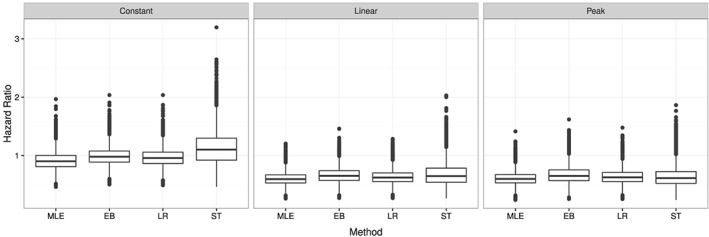
Boxplot of estimated hazard ratios at the interim analysis in the three scenarios for *K*=4. MLE, maximum likelihood estimator; EB, EB shrinkage estimator; LR, LR shrinkage estimator; ST, Stallard–Todd estimator.

While the estimates of the Stallard–Todd method are on average not far from those of the other methods, a closer look at the distribution of the estimated HRs at the interim analysis (Figure 3) reveals that the Stallard–Todd method produces more extreme and skewed results, than the other methods, especially in the “Constant” scenario.

**Figure 4:**
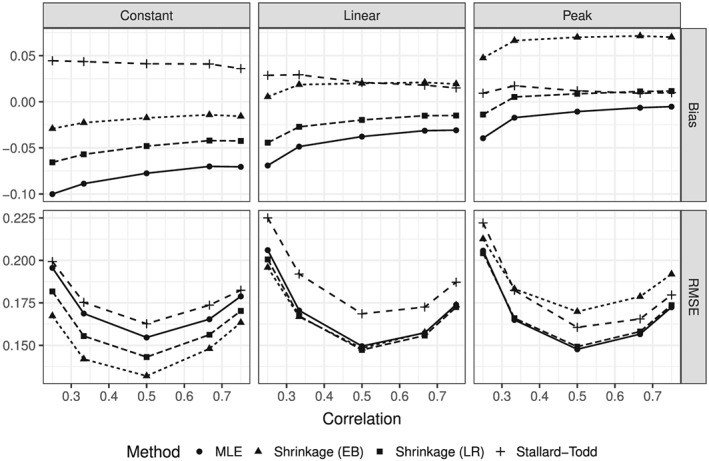
Bias and Root mean squared error (RMSE) of the two‐stage estimators as functions of the correlation for *K*=4 treatment groups in each of the three base scenarios.

The influence of the allocation ratio on the bias and MSE of the two‐stage estimators is shown in Figure 4. A maximum of *n* patients were allocated to each treatment group and a maximum of *m* patients to the control group. The allocation ratios *m*:*n* considered were 3:1, 2:1, 1:1, 1:2, and 1:3. This corresponds to correlations between the estimated log‐HRs of approximately 1/4, 1/3, 1/2, 2/3, and 3/4. The bias increases with increasing correlation in line with our theoretical result. The MSE apparently attains its minimum at a correlation of 0.5. This suggests that patients should be equally allocated to all groups.

**Figure 5:**
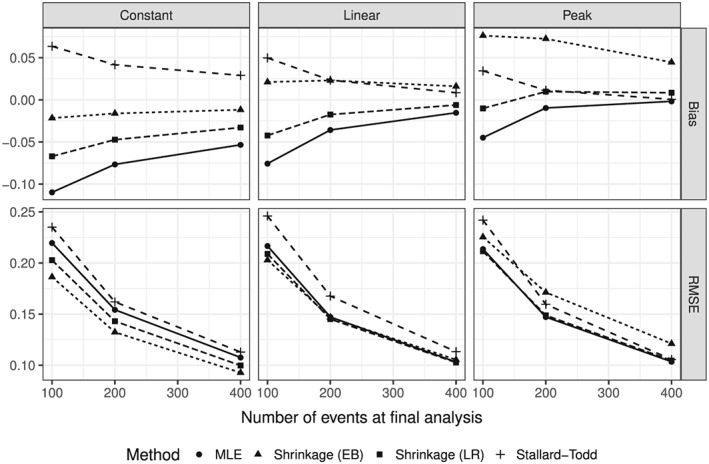
Bias and Root mean squared error (RMSE) of the two‐stage estimators as functions of the number of events at the final analysis for *K*=4 treatment groups in each of the three base scenarios.

The bias and MSE decrease when the number of observed events increases (Figure 5). There is a considerable finite sample bias in addition to the selection bias, when only a few events (<25 events per group) are observed.

The largest effect considered is a HR of 0.6. The MLE is already practically unbiased when the HR is 0.6 for one treatment and 1 for all other treatments as can be seen in Figure 1. An even larger effect would not provide any additional insight, although estimation also works well for values smaller than 0.6.

## 4 Application

We apply our methods to data reconstructed from the published Kaplan–Meier curves of the FOCUS trial 13 using the method of 27. The Kaplan–Meier curves were digitized using WebPlotDigitizer 28.

In the FOCUS trial, five treatment strategies of sequential and combination chemotherapy for poor prognosis advanced colorectal cancer were compared: flourouracil only (arm A, control), irinotecan after flourouracil (arm B‐ir), combination of flourouracil and irinotecan (arm C‐ir), oxaliplatin after flourouracil (arm B‐ox), and combination of flourouracil and oxaliplatin (arm C‐ox). A total of 2135 patients were randomized in the ratio 2:1:1:1:1.

The reconstructed data closely match the original data in terms of hazard ratios and corresponding confidence intervals (Table 1). The data are analyzed under a hypothetical two‐stage design, with an interim analysis after 900 events across all arms. This corresponds to roughly 300 events in the control arm and 150 events in each of the four treatment arms. The final analysis is conducted after 900 events in the control arm and the selected treatment arm, resulting in about 600 events in the control arm and 300 events in the selected treatment arm. In addition to point estimates, bootstrap confidence intervals from 1000 replications for all methods conditional on the treatment selection in the original data set are provided. The resulting confidence intervals therefore do not account for the variability introduced by the selection and are expected to have less than the nominal coverage as discussed in Section 2.3.

**Table 1:** HRs, 95% confidence intervals, and *p*‐values of the original FOCUS data 13 and the reconstructed data.

	Original data		Reconstructed data	
Comparison	HR (95% CI)	*p*‐value	HR (95% CI)	*p*‐value
A versus B‐ir	0.91 (0.79–1.03)	0.16	0.91 (0.79–1.05)	0.18
A versus C‐ir	0.84 (0.73–0.96)	0.01	0.84 (0.73–0.97)	0.02
A versus B‐ox	0.97 (0.85–1.11)	0.65	0.96 (0.84–1.10)	0.58
A versus C‐ox	0.93 (0.81–1.06)	0.26	0.91 (0.79–1.05)	0.18

HR, hazard ratio.

Both shrinkage methods produce very similar and sensible results (Table 2), when compared with the original observed HRs of the FOCUS trial (Table 1), where no selection was performed. The estimated HRs of the LR shrinkage method are very close the MLE in all arms, with narrower confidence intervals, while the EB shrinkage method shrinks all estimates to the overall HR, resulting in a smaller treatment effect in the selected C‐ir arm. This is the same tendency of the EB shrinkage estimator to overcorrect the bias that was also observed in the “Linear” and “Peak” simulation scenarios.

**Table 2:** Estimated HRs and 95% bootstrap confidence intervals for all treatment groups (versus A).

		Hazard ratio (95% CI)		
Method	Group	Interim analysis	Final analysis	Two‐stage
MLE	B‐ir	0.87 (0.69–1.04)	0.91 (0.80–1.04)	0.91 (0.80–1.04)
	C‐ir	0.79 (0.63–0.94)	0.84 (0.73–0.97)	0.84 (0.73–0.97)
	B‐ox	0.88 (0.72–1.05)	0.96 (0.83–1.10)	0.96 (0.83–1.10)
	C‐ox	0.81 (0.66–0.98)	0.91 (0.79–1.04)	0.91 (0.79–1.04)
EB	B‐i	0.84 (0.72–0.97)	0.90 (0.82–1.01)	0.89 (0.80–1.02)
	C‐ir	0.84 (0.68–0.94)	0.90 (0.77–0.99)	0.87 (0.76–0.98)
	B‐ox	0.84 (0.72–0.99)	0.90 (0.83–1.05)	0.94 (0.83–1.07)
	C‐ox	0.84 (0.70–0.95)	0.90 (0.81–1.02)	0.92 (0.81–1.04)
LR	B‐ir	0.84 (0.71–1.02)	0.91 (0.80–1.03)	0.90 (0.80–1.03)
	C‐ir	0.83 (0.64–0.94)	0.87 (0.75–0.98)	0.86 (0.74–0.97)
	B‐ox	0.85 (0.72–1.03)	0.94 (0.83–1.08)	0.94 (0.83–1.09)
	C‐ox	0.83 (0.68–0.97)	0.91 (0.80–1.03)	0.92 (0.80–1.04)
ST	B‐ir	0.86 (0.61–1.03)	–	0.91 (0.75–1.04)
	C‐ir	0.96 (0.64–1.58)	–	0.93 (0.75–1.24)
	B‐ox	0.88 (0.65–1.05)	–	0.96 (0.81–1.10)
	C‐ox	0.72 (0.55–0.98)	–	0.86 (0.73–1.04)

Interim analysis at 50% of the total number of events. C‐ir was selected at interim. Follow‐up continued in all groups until the final analysis.

MLE, maximum likelihood estimator; EB, EB shrinkage estimator; LR, LR shrinkage estimator; ST, Stallard–Todd estimator.

The Stallard–Todd estimator applied to this specific data set at the interim analysis gives rather different results, which contradict the observed HRs in the original data, where the C‐ir arm was the best performing treatment arm. While this result is unsatisfactory, it is in‐line with the simulation results (Figure 3) that show that extreme solutions can be obtained with this approach. The iterative procedure did not converge when applied at the final analysis (see also Section 2.4).

The effect of continuing follow‐up in the dropped treatment arms can be seen clearly in the B‐ir and C‐ox arms. The substantial difference in Stage 1 HRs completely disappears at the end of Stage 2.

## 5 Conclusion

The bias of the MLE was found to be small except for a large number ( K⩾6) of treatment groups. We have shown that the common control group reduces the bias of the MLE because of the induced correlation of the MLEs and that the bias of the MLE in the dropped treatment arms is reduced when continuing follow‐up until the final analysis in accordance with the simulation results of 23.

In our simulation study, the shrinkage methods performed very well, when all treatment effects are equal, but had a larger bias than the MLE, which increased with number of treatment groups, when the the maximal absolute difference of the true treatment effects was large. These results reflect the prior assumptions underlying the empirical Bayes estimators. When comparing the estimators with respect to the MSE, the Stallard–Todd method performs the worst across all scenarios. This is also reflected in the analysis of the reconstructed FOCUS trial data, which shows that either shrinkage method is preferable to the Stallard–Todd method.

Unless there is reason to believe that there are large differences between the true treatment effects, the LR shrinkage estimator can be recommended as a way to reduce the bias and the MSE of the MLE. Because the shrinkage estimators tend to overcorrect the bias, they might also be of interest in situations where overestimation of the treatment effect is considered worse than underestimation.

All methods were based on the asymptotic normal approximation of the log‐HRs. In our main simulations, the number of events was at least 150, such that finite sample bias is negligible. This can be seen, for example, in the Peak scenario in Figure 1, where the MLE is practically unbiased. However, when the number of events per group is very small (<25), the finite sample bias outweighs the selection bias as was seen in Figure 5. Such small event numbers do not seem relevant in practice, because typically much larger event numbers are required to achieve acceptable power.

Assuming a Cox proportional hazards model is convenient but not necessary, because all methods are based on the asymptotic approximation of the distribution of the treatment effect estimators. Therefore, the Cox model could be replaced with any other model with asymptotically normal treatment effect estimators. It is anticipated that the results would be comparable to those under the Cox model.

Confidence intervals after adaptive treatment selection that have the correct coverage probability are non‐trivial and were outside of the scope of this work. As far as we know, no adequate methods for construction of confidence intervals for the shrinkage or Stallard–Todd methods have been proposed.

In most trials some form of covariate adjustment is required, for example, inclusion of center effects in a multi‐center trial to account for stratified randomization. As long as the asymptotic results still hold, no adjustment is necessary, because only the dimension *K* of the multivariate normal distribution needs to be increased to accommodate any additional regression coefficients.

The shrinkage estimators could also be derived as estimators arising from penalized Cox regression 29. When using an *L*_2_ penalty, that is, $\sum_{k=1}^{K}{\left(\beta_{k}-\bar{\beta }\right)}^{2}$, there is a one‐to‐one correspondence between the penalty parameter and the prior variance *τ*. This is appealing, because the penalized Cox regression works directly on the time‐to‐event data and not only via an asymptotic normal approximation. Selection of the penalty parameter could be performed by cross‐validation in order to minimize the prediction MSE. However, cross‐validation is computationally extremely expensive and results in an additional cross‐validation error. In simulations not reported here, the penalized MLE with cross‐validation did not perform better than the shrinkage estimator.

The LR shrinkage of the vector of MLEs does not change the ordering of its components, because

$${\hat{\beta }}_{k}^{LR,1}-{\hat{\beta }}_{l}^{LR,1}={Ĉ}^{+}\left({\hat{\beta }}_{k}^{\text{MLE}\;,1}-{\hat{\beta }}_{l}^{\text{MLE}\;,1}\right)\;(1⩽k,l⩽K)$$

and ${Ĉ}^{+}⩾0$. Thus, the results would be the same if selection were based on the LR shrinkage estimator instead of the MLE.

Finally, it is worth mentioning that the calculation of the shrinkage estimator does not depend on the specific selection rule used. It can be used without change with complex selection rules, for example, when selection is based on hypothesis tests. For the Stallard–Todd method and the UMVCUE approach, non‐trivial modifications are necessary 20.

## Appendix A Asymptotic covariance matrix of $\hat{\beta }$ ^MLE^ (t)

### A.1

Weak convergence of the maximum likelihood estimator (MLE) as a function of calendar time to a mean‐zero Gaussian field {η(t):t⩾0} follows from theorem 4.2 of 30. For t⩾0, define the matrix *μ*(*t*) by

$$\mu_{kj}(t)=I\{k=j\}p_{k}e^{\beta_{k}}\int_{0}^{t}S_{k}(t,u)\lambda_{0}(u)du-p_{k}p_{j}e^{\beta_{k}+\beta_{j}}\int_{0}^{t}\frac{S_{k}(t,u)S_{j}(t,u)}{\overset{K}{\underset{l=0}{\sum }}p_{l}S_{l}(t,u)e^{\beta_{l}}}\lambda_{0}(u)du\;(1⩽k,j⩽K),$$

where *S*_*k*_(*t*, *u*) = *P*(*T*∧*C*∧(*t*−*R*)^+^>*u*|*Z*_*k*_ = 1),*S*_0_(*t*,*u*) = *P*(*T*∧*C*∧(*t*−*R*)^+^>*u*|*Z*_1_ = … = *Z*_*K*_ = 0) and *β*_0_ = 0. Then, for *j* = 1, 2,

$$\sqrt{n}\left\{{\hat{\beta }}^{\text{MLE}\;,j}(\cdot )-\beta \right\}\overset{L}{\to }\eta (\cdot )\;(n\to \infty ),$$

where the covariance function of *η* is given by

$$E\left[\eta {\left(s\right)}^{\prime }\eta (t)\right]=\mu^{-1}(s\vee t)\;(s,t⩾0).$$

For *β*≈0 and *C* independent of *Z*, the asymptotic covariance matrix of $\sqrt{n}\left\{{\left({\hat{\beta }}_{1}^{\text{MLE}\;,j}(t),{\hat{\beta }}_{2}^{\text{MLE}\;,j}(t)\right)}^{T}-{\left(\beta_{1},\beta_{2}\right)}^{T}\right\}$ is the inverse of the matrix:

$$\tilde{\mu }(t)=\left(\begin{array}{cc} {\tilde{p}}_{1}(1-{\tilde{p}}_{1}) & -{\tilde{p}}_{1}{\tilde{p}}_{2}\\ -{\tilde{p}}_{1}{\tilde{p}}_{2} & {\tilde{p}}_{2}(1-{\tilde{p}}_{2}) \end{array}\right)\int_{0}^{t}S_{0}(t,u)\lambda_{0}(u)du,$$

where ${\tilde{p}}_{i}=p_{i}/(p_{0}+p_{1}+p_{2})$. Consequently, we have for the correlation *ρ*_12_(*t*) between ${\hat{\beta }}_{1}^{\text{MLE}\;,j}(t)$ and ${\hat{\beta }}_{2}^{\text{MLE}\;,j}(t)$:

$$\rho_{12}(t)\approx \frac{{\tilde{\mu }}_{12}^{-1}(t)}{\sqrt{{\tilde{\mu }}_{11}^{-1}(t)}\sqrt{{\tilde{\mu }}_{22}^{-1}(t)}}=\frac{p_{1}p_{2}}{\sqrt{p_{0}+p_{1}}\sqrt{p_{0}+p_{2}}}.$$

## Appendix B Proofs

### B.1

#### B1 Selection probability

(B.1) $$\begin{array}{ccc} P(S=k)=\; & P\left({\hat{\beta }}_{k}^{\text{MLE}\;,1}=\underset{j}{\min}{\hat{\beta }}_{j}^{\text{MLE}\;,1}\right) & \\ =\; & E\left[P\left(\underset{j\neq k}{\cap }\left\{{\hat{\beta }}_{j}^{\text{MLE}\;,1}>x\right\}|{\hat{\beta }}_{k}^{\text{MLE}\;,1}=x\right)\right]\\ = & \int_{-\infty }^{\infty }S_{\beta_{-k}(x),\Sigma_{-k}(x)}(x)\phi \left(\frac{x-\beta_{k}}{\sigma_{k}}\right)\sigma_{k}^{-1}dx. \end{array}$$

If ${\hat{\beta }}_{k}^{\text{MLE}\;,1}$ (*k* = 1, …, *K*) are independent, then

$$P(S=k)=\Phi_{\beta_{-k}-\beta_{k},{\tilde{\Sigma }}_{k}}(0,\ldots ,0),$$

where ${\tilde{\Sigma }}_{k}$ is the covariance matrix of ${\hat{\beta }}_{-k}^{\text{MLE}\;,1}-{\hat{\beta }}_{k}^{\text{MLE}\;,1}$.

#### B2 Conditional selection bias

##### B.2.1 At the interim analysis

At the interim analysis, the selection bias of ${\hat{\beta }}_{k}^{\text{MLE}\;,1}$ conditional on *S*=*k* is

(B.2) $$\begin{array}{ccc} {\text{cb}}_{1k}^{+}=\; & \frac{E\left[\left({\hat{\beta }}_{k}^{\text{MLE}\;,1}-\beta_{k}\right)I\left\{S=k\right\}\right]}{P(S=k)} & \\ =\; & \frac{E\left[\left({\hat{\beta }}_{k}^{\text{MLE}\;,1}-\beta_{k}\right)P\left(S=k|{\hat{\beta }}_{k}^{\text{MLE}\;,1}\right)\right]}{P(S=k)}. \end{array}$$

Equation (2) now follows from ${\hat{\beta }}_{k}^{\text{MLE}\;,1}∼N(\beta_{k},\sigma_{k}^{2})$ and

$$P(S=k|{\hat{\beta }}_{k}^{\text{MLE}\;,1}=x)=P\left({\hat{\beta }}_{l}^{\text{MLE}\;,1}>x\;\forall l\neq k|{\hat{\beta }}_{k}^{\text{MLE}\;,1}=x\right)=S_{\beta_{-k}(x),\Sigma_{-k}(x)}(x).$$

In the dropped treatment arm, the conditional bias of ${\hat{\beta }}_{k}^{\text{MLE}\;,1}$ given *S*≠*k* is

(B.3) $$\begin{array}{ccc} {\text{cb}}_{1k}^{-}=\; & \frac{E\left[{\hat{\beta }}_{k}^{\text{MLE}\;,1}(1-I\{S=k\})\right]-\beta_{k}(1-P(S=k))}{1-P(S=k)} & \\ = & -\left(E\left[{\hat{\beta }}_{k}^{\text{MLE}\;,1}|S=k\right]-\beta_{k}\right)\frac{P(S=k)}{1-P(S=k)}. \end{array}$$

##### B.2.2 At the final analysis

We have that

$$E\left[{\hat{\beta }}_{k}^{\text{MLE}\;,2}|{\hat{\beta }}_{1}^{\text{MLE}\;,1},\ldots ,{\hat{\beta }}_{K}^{\text{MLE}\;,1}\right]=\beta_{k}+u_{k}\left({\hat{\beta }}^{\text{MLE}\;,1}-\beta \right).$$

Therefore,

(B.4) $$\begin{array}{ccc} {\text{cb}}_{2k}^{+}= & \frac{E\left[E\left[{\hat{\beta }}_{k}^{\text{MLE}\;,1}|{\hat{\beta }}_{1}^{\text{MLE}\;,1},\ldots ,{\hat{\beta }}_{K}^{\text{MLE}\;,1}\right]I\{S=k\}\right]}{P(S=k)}-\beta_{k} & \\ = & \overset{K}{\underset{l=1}{\sum }}u_{kl}v_{kl}. \end{array}$$

In the dropped treatment arm, the conditional bias of ${\hat{\beta }}_{k}^{\text{MLE}\;,2}$ given *S*≠*k* is

$${\text{cb}}_{2k}^{-}=-\left(E\left[{\hat{\beta }}_{k}^{\text{MLE}\;,2}|S=k\right]-\beta_{k}\right)\frac{P(S=k)}{1-P(S=k)}.$$
